# Endocannabinoid response to social stress in chronic non-medical prescription opioid users

**DOI:** 10.1007/s00213-025-06950-4

**Published:** 2025-11-05

**Authors:** Vinzenz K. Schmid, Boris B. Quednow, Daniele Pellegata, Philip Meier, Jürg Gertsch, Sara L. Kroll

**Affiliations:** 1 Social and Affective Neuropsychopharmacology, Department for Adult Psychiatry and Psychotherapy, University Hospital of Psychiatry Zurich, University of Zurich, Lenggstrasse 31, Zurich, 8032 Switzerland; 2 Experimental Pharmacopsychology and Psychological Addiction Research, Department for Adult Psychiatry and Psychotherapy, University Hospital of Psychiatry Zurich, University of Zurich, Zurich, Switzerland; 3 Neuroscience Center Zurich, University of Zurich and Swiss Federal Institute of Technology, Zurich, Switzerland; 4 Institute of Biochemistry and Molecular Medicine, University of Bern, Bern, Switzerland

**Keywords:** Addiction, Opiates, Morphin, Fentanyl, Oxycodone, Endocannabinoids, Endocannabinoid system, 2-arachidonoylglycerol, Rejection, Ostracism

## Abstract

**Rationale:**

The ongoing global opioid crisis underscores the need to better understand the neurobiological mechanisms underlying opioid use disorder (OUD). Stress is a key risk factor for developing and maintaining OUD. While animal models report that the endocannabinoid system (ECS) plays a modulatory role in stress response, alterations of ECS stress reactivity in individuals with OUD have yet to be studied.

**Objectives:**

Here, we aimed to study the response of the ECS to experimentally-induced mild psychosocial stress in individuals with non-medical prescription opioid use (NMPOU) without intravenous use.

**Methods:**

We compared plasma concentrations of the two main endocannabinoids 2-arachidonoylglycerol (2-AG) and anandamide (AEA) along with structurally related *N*-acylethalonamines (NAEs) and arachidonic acid (AA) between individuals with chronic NMPOU (*n* = 21) and matched opioid-naïve healthy controls (*n* = 29) after social exclusion using the Cyberball task. Blood samples were collected before stress induction, and 10, 20, 30, and 60 min after stress onset.

**Results:**

We found a significant GROUP*TIME interaction for 2-AG, with controls showing increased 2-AG plasma levels after stress, contrasting with a blunted stress response in NMPOU. Both groups robustly differed in 2-AG levels at all time points after stress induction. No significant GROUP*TIME interactions were found for AEA, NAEs, and AA. Increased 2-AG levels were associated with greater *feelings of social inclusion* overall.

**Conclusion:**

Results suggest dysfunctional stress response at the level of the ECS in individuals with NMPOU. Specifically 2-AG might play a critical role in stress resilience and, thus, it might be a potential pharmacotherapeutic target in the treatment of OUD.

**Supplementary Information:**

The online version contains supplementary material available at 10.1007/s00213-025-06950-4.

## Introduction

The opioid crisis continues to increase worldwide, with North America at the epicenter of this public health emergency. Opioid-related overdose deaths accounted for over 80% of all drug-related fatalities in the United States (Centers for Disease Control and Prevention 2024) and more than doubled within the past five years in Canada (Public Health Agency of Canada 2024). Also in Europe, regions like Scotland have reported comparable trends underscoring the global reach of this crisis (Amsterdam et al. 2021). Despite decades of research, knowledge gaps persist in understanding the neurobiological mechanisms underpinning opioid use disorder (OUD). This is particularly reflected in a lack of treatment approaches achieving opioid abstinence. Opioid substitution therapy (OST) with methadone or buprenorphine remains the primary treatment for OUD, mitigating opioid craving and withdrawal symptoms, while maintaining opioid dependence (Lee et al. 2024). However, many patients continue using illicit opioids during OST (Zhu et al. 2018; Moses et al. 2022), and relapse rates after detoxification as well as discontinuation of OST remain high (Smyth et al. 2010; Weiss et al. 2011; Bentzley et al. 2015). These challenges highlight the urgent need for a better understanding of the underlying neurobiological mechanisms underpinning chronic opioid use to improve prevention and treatment outcome for OUD (Blanco and Volkow 2019; Strain et al. 2021; Lee et al. 2022).

Stress is discussed as a key etiological factor in all substance use disorders (SUD), with evidence indicating that stress can contribute to both, the initiation, development, and maintenance of SUD (Koob and Volkow 2016; Sinha 2024). In turn, substance use itself can alter stress perception and physiological stress responses, creating the vicious cycle of drug addiction (Wemm and Sinha 2019). Animal models have shown that acute pharmacological manipulation of the µ-opioid receptor (MOR) system modulates stress responses. In particular, activation of the MOR via opioids, such as morphine, has dampening stress effects, especially in social contexts, while the blockade of MOR by opioid antagonists, such as naltrexone, produced the opposite effect (Herman and Panksepp 1978; Bali et al. 2015). In addition to the well known dampening effect of acute opioid administration on the hypothalamic-pituitary-adrenal (HPA) axis (McDonald et al. 1959; Massaccesi et al. 2022), we previously showed dysfunctional stress response of the HPA axis after the stressor of social exclusion in individuals with chronic non-medical prescription opioid use (NMPOU) compared to healthy controls (Kroll et al. 2018).

Preclinical evidence indicates that the endocannabinoid system (ECS) is crucially involved in regulation of stress processes (Steiner and Wotjak 2008; Finn 2010; Morena et al. 2016). The two main endocannabinoids anandamide (AEA) and 2-arachidonoylglycerol (2-AG) have been linked to immediate stress responses and termination of stress reactivity, respectively, by stimulating cannabinoid 1 (CB1) receptors in brain regions involved in stress regulation, such as the basolateral amygdala and prefrontal cortex (Hill et al. 2011; deRoon-Cassini et al. 2020; Scheyer et al. 2022). Particularly CB1 receptors can regulate the HPA axis and sympathetic nervous system (SNS), as their activation mitigates acute stress responses (Maldonado et al. 2020). Animal models consistently showed a rapid reduction in AEA in the basolateral amygdala in response to a stressor (Patel et al. 2005; Hill et al. 2009a; Gray et al. 2015), followed by a delayed increase in 2-AG in the paraventricular nucleus of the hypothalamus and the medial prefrontal cortex (Evanson et al. 2010; Hill et al. 2011). In contrast, the limited human studies available reported mixed findings, with some showing decreased AEA levels following acute stress (Mayo et al. 2018, 2020), others reporting an increase (Dlugos et al. 2012; Crombie et al. 2019), and some finding no changes in AEA levels (Hill et al. 2009b; Ney et al. 2021). Similarly, while some studies observed increased 2-AG levels post-stressor (Hill et al. 2009b; Crombie et al. 2019; Ney et al. 2021), others found no significant changes (Dlugos et al. 2012; Mayo et al. 2018, 2020). These mixed results are probaly caused by methodological differences between human studies such as using different stress tasks, biological samples for endocannabinoid analyses (serum, plasma, saliva), sample processing (cooling, etc.), and sampling time points.

Animal models suggest a specific interaction between the ECS and the endogenous opioid system (Fattore et al. 2005; Robledo et al. 2008; Lopez-Moreno et al. 2010). In particular, the CB1 receptor and MOR are often co-localized in the brain resulting in synergistic inhibitory effect on neurotransmitter release (Fattore et al. 2005; Robledo et al. 2008). Acute opioid administration can influence endocannabinoid signaling, reporting increased AEA and decreased 2-AG levels in reward-related brain areas (Viganò et al. 2004; Caillé et al. 2007), potentially contributing to the development of opioid dependence (Robledo et al. 2008; Aghaei et al. 2023). We recently reported elevated basal *N*-acylethalonamine (NAE) levels, including AEA, in individuals with chronic non-medical prescription opioid use (NMPOU), while individuals with cocaine dependence showed elevated 2-AG plasma levels compared to healthy controls and recreational cocaine users (Kroll et al. 2023, 2024).

A dysfunctional and stronger stress response has been reported in individuals with OUD compared to healthy controls (MacLean et al. 2019). However, little is known about stress response of the ECS in individuals with chronic opioid use. Therefore, we aimed at investigating stress reactivity of the ECS to a laboratory induced social stressor in NMPOU compared to healthy controls. We examined stress induced changes of endocannabinoids and related lipids after social exclusion, using the Cyberball task, at five time points: baseline, 10, 20, 30, and 60 min after stress onset. In our previous study using the same sample, individuals with NMPOU showed elevated adrenocorticotropic hormone (ACTH) and cortisol responses to social exclusion compared to controls, whereas sympathetic reactivity and subjective stress response were not different between groups (Kroll et al. 2019). These findings indicate a dysfunctional stress reactivity of the HPA axis in individuals with chronic NMPOU. Given the reported involvement of AEA and 2-AG in stress regulation particularly modulated by glucocorticoids (Mayo et al. 2020; deRoon-Cassini et al. 2020; Spohrs et al. 2022), we hypothesized that individuals with NMPOU would exhibit a dysfunctional stress response also at the level of the ECS. More precisely, we expected altered circulating levels of AEA and 2-AG after stress induction by social exclusion in individuals with NMPOU compared to controls. For this, we expanded on our previous study showing elevated basal NAE levels in NMPOU compared to controls, associated with less feelings of social exclusion (Kroll et al. 2024). Here, we investigated alterations in stress reactivity of NAEs and arachidonic acid (AA) between groups as well as potential correlations between the ECS stress response and previously reported subjective as well as objective stress reactivity measures (Kroll et al. 2019).

## Methods

### Participants

The present secondary analyses were conducted using data and biological samples from a previously published study that investigated stress reactivity of the HPA axis in response to social rejection (Kroll et al. 2019). Initially, 25 individuals with NMPOU and 30 matched opioid-naïve controls were recruited. One control participant was excluded due to non-compliance during cognitive testing. Two individuals with self-reported NMPOU were excluded due to negative toxicology results in both hair and urine samples. This resulted in a final sample of 23 NMPOU participants and 29 controls. For the current analyses of endocannabinoids, we included participants with available plasma samples, resulting in 21 individuals with NMPOU and 29 controls. All participants were recruited via advertisements posted in internet forums, local newspapers, and specialized addiction treatment centers.

Inclusion criteria for the NMPOU group required chronic non-medical prescription opioid use over at least the past six months, confirmed by toxicological analysis of 6 cm long hair samples (for technical details, see (Kroll et al. 2018). Participants were excluded if they had a current or past history of intravenous street heroin use or heroin dependence. More detailed information on inclusion and exclusion criteria is provided elsewhere (Kroll et al. 2018); briefly, exclusion criteria covered neurological disorders or head injuries, severe physical diseases, frequent cannabis use, severe psychiatric disorders such as post-traumatic stress disorder (exceptions were made for alcohol or tobacco use disorders and past depressive episodes), chronic pain, and recent emotionally stressful or painful events.

They were instructed to abstain from all psychotropic substances for 72 h prior to testing and from alcohol for 24 h before the start of the test session. Individuals in the NMPOU group were additionally asked to refrain from opioid use on the day of testing or, if necessary, to take only a minimal dose sufficient to prevent withdrawal symptoms with the intention of avoiding measuring acute or withdrawal effects. Substance use in the last days was monitored by urine testing at the start of the session.

The study was approved by the Cantonal Ethics Committee of Zurich (KEK-Nr. 2015 − 0238) and was conducted in accordance with the Declaration of Helsinki. All participants provided written informed consent. They received financial compensation for their participation.

### Study procedure and measurements

Testing started at approximately 11am to account for potential circadian influences in neuroendocrine measures. Participants were initially screened for psychiatric disorders, using the *Structured Clinical Interview for DSM-IV Disorders* (SCID-I; (Wittchen et al. 1997), which was adapted for DSM-5 SUD criteria. Participants then completed the standardized *Interview for Psychotropic Drug Consumption* (Quednow et al. 2004) to document their substance use history. Eligible participants underwent further screening: nicotine dependence was measured with the *Fagerström Test for Nicotine Dependence* (FTND; (Heatherton et al. 1991), depressive symptoms were evaluated via the *Beck Depression Inventory* (BDI; (Beck et al. 1961), and premorbid verbal intelligence quotient (IQ) was estimated using a German vocabulary test (Lehrl 1999). Additional opioid-related measures assessed in the NMPOU group included current opioid craving using a Numeric Rating Scale (NRS) from 1 (no craving) to 10 (highest craving), time since last opioid use (hours), years of use, weekly opioid use converted to morphine equivalents (ME) in mg (for ME conversion details see (Kroll et al. 2018), and withdrawal symptoms assessed by the *Objective Opioid Withdrawal Scale* (OOWS; (Handelsman et al. 1987). Furthermore, opiod concentrations in hair samples were also transformed into ME (pg/mg) providing an objective standardized measure of long-term opioid use over the last six months. Following these assessments, an intravenous catheter was inserted into the forearm vein of each participant’s non-dominant hand and a whole blood sample was taken. After a one-hour resting period, a baseline plasma sample was obtained 20 min before starting the social exclusion task (T1). Subsequent samples were then collected 10 min (T2), 20 min (T3), 30 min (T4), and 60 min (T5) after the task’s onset, allowing for assessment of changes in the plasma profiles related to psychosocial stress (Kirschbaum et al. 1999). Participants completed further neuropsychological tests after the social exclusion task, as reported in previous publications (Kroll et al. 2018, 2021).

### Social stress task (Cyberball) and stress measures

The Cyberball task is a computer-based paradigm that reliably induces negative affect and feelings of social exclusion (Zadro et al. 2004; Williams and Jarvis 2006; Hartgerink et al. 2015). To enhance credibility of the task, participants were introduced to two other players in person and images of all players were displayed on the screen during the task. This virtual ball-tossing game, entirely computer-controlled, consisted of 60 tosses over a total duration of three minutes. During the first minute, participants were included in the game and received the ball six times (10%). For the remaining two minutes, participants were systematically excluded from the game and no longer received the ball, simulating social rejection.

Subjective and physiological stress responses to the Cyberball task of this study sample were reported previously (Kroll et al. 2019). In brief, subjective stress responses to social rejection were assessed using the *Positive and Negative Affect Schedule* (PANAS; (Watson et al. 1988) administered before and after the task, with change scores used in the analyses. Following the task, participants rated their perceived levels of inclusion, exclusion, and estimated percentage of ball receptions on a nine-point Likert scale.

For physiological stress responses, we assessed skin conductance using spontaneous fluctuations (SF) as a proxy for SNS activity. SF were analyzed using a dynamic causal model (DCM), which employs a non-linear estimation approach with a detection threshold of 0.1 µS, allowing for a precise and sensitive estimation of sympathetic activation (Bach et al. 2011). Heart rate (HR) was measured throughout the Cyberball task via a three-lead electrocardiogram (ECG) recorded at 1000 Hz. Additionally, root mean square of successive differences in heart rate variability (RMSSD-HRV) was assessed as an index of vagally-mediated parasympathetic activity (PNS) (Laborde et al. 2017). Stress reactivity of the HPA axis was measured by plasma cortisol and ACTH analyses and calculation of the area under the curve with respect to increase (AUC_i_) based on Pruessner et al. (2003). For a detailed description of the neuroendocrine assessment, physiological data acquisition, and analysis methods, see Kroll et al. (2019).

### Quantification of endocannabinoids, NAEs, and AA

All blood samples were collected using BD Vacutainer^®^ lithium-heparin tubes (6.0 ml; no gel), gently inverted 10–15 times immediately after filling, and placed in an insulated 4 °C ice bath for a maximum of 85 min prior to processing. Five minutes after the last blood draw, samples were centrifuged in a pre-cooled centrifuge with a swing-bucket rotor (Universal 320 R, Hettich) at 1300×g for 10 min at 4 °C. Plasma was separated immediately, three 500 µl aliquots were transferred into Eppendorf 1.5 ml Safe-Lock tubes and stored within 5 min of centrifugation at − 80 °C to preserve sample integrity for subsequent analyses. Plasma levels of the main endocannabinoids AEA and 2-AG, the NAEs oleoylethanolamide (OEA), palmitoylethanolamide (PEA), linoleoyl ethanolamide (LEA), and stearoyl ethanolamide (SEA), as well as AA were quantified using liquid-liquid extraction followed by liquid chromatography-electrospray ionization-tandem mass spectrometry (LC-ESI-MS/MS) analysis (Reynoso-Moreno et al. 2021). For a detailed description see Supplementary Materials Method S1. SEA was excluded from analyses due to levels falling below the limit of quantification in several participants. Plasma samples at the last time point (T5) were missing for one control and two individuals with NMPOU. To keep these cases in our AUC_i_ analyses and enhance statistical power, we replaced each missing with the last available observation prior to it (Last Observation Carried Forward).

Values of endocannabinoids, NAEs, and AA plasma concentrations were winsorized within each group to account for outliers while retaining the rank order of data (Wilcox 2011).

### Statistical analyses

The primary objective of our analyses was to examine whether laboratory stress induction elicited differential changes in circulating endocannabinoids between NMPOUs and controls. Our statistical approach was designed to (1) model changes across multiple repeated measurements, while controlling for known confounding variables, (2) summarize overall stress response, and (3) explore associations with subjective and physiological stress measures.

All statistical analyses were performed using IBM SPSS Statistics (Version 29.0.0.0).

To assess demographic variables between groups, Pearson’s chi-squared (X^2^) tests were used for categorical variables (Pearson 1900). For continuous variables violating normality assumptions, the Mann-Whitney U test was applied as a non-parametric alternative to the t-test (Mann and Whitney 1947). For normally distributed continuous variables, independent-samples t-tests were used.

1. Main analyses: linear mixed models.

Linear mixed models (LMMs) for repeated measures were performed for our main outcome to assess stress response of endocannabinoids AEA and 2-AG as well as for the exploratory analyses of changes in NAEs and AA concentrations after stress induction. LMMs are robust to outliers and missing values while accounting for within-subject correlations and allowing flexible modeling of time effects. The models included *GROUP* (control, NMPOU), *TIME* (five time points), and their interaction GROUP*TIME as fixed effects, with *SUBJECTS* as random intercepts to capture individual baseline differences. A first-order autoregressive covariance structure was chosen to reflect higher correlation for adjacent time points.

To control for well-known confounding variables influencing the ECS, we included *AGE* (Piyanova et al. 2015) and *SEX* (Vecchiarelli et al. 2022) into all of the models, as well as *CANNABIS USE* within the last six months (yes/no), given that cannabis use might broadly affect the ECS (Boachie et al. 2023). In models showing significant GROUP*TIME interactions, we further tested potential influences of depression (*BDI* sum score), *BMI*, *SMOKING* within the last six months (yes/no), and *ALCOHOL USE* within the last six months (yes/no) as separate predictors, together with *AGE* and *SEX*. Post-hoc comparisons were performed using dependent (for within-group time changes) and independent sample t-tests (for between-group differences at specific time points).

2. AUC_i_ analyses.

To provide a single index of ECS stress reactivity, we calculated the AUC_i_ of endocannabinoids, NAEs, and AA. Group differences in AUC_i_ were analyzed using independent-sample t-tests, and analyses of covariance (ANCOVA) were used to control for *SEX*,* AGE*, and *CANNABIS USE* as reported for the LMMs before. Multiple linear regression models (forced entry) were performed to identify further potential confounding variables such as *BDI*,* BMI*,* SMOKING*, and *ALCOHOL USE* (as reported above), and added as covariates into the ANCOVAs, if significant (Maldonado and Greenland 1993).

3. Correlation analyses.

Additional Spearman’s rank correlation analyses were conducted for outcomes showing significant group differences in AUC_i_ to examine potential associations with subjective as well as physiological stress measures. Furthermore, correlations between opioid use variables and endocannabinoids were assessed within the NMPOU group. Robustness of significant correlations was calculated by performing bootstrapping with a number of 1000 resamples.

Effect sizes were calculated as Cohen’s *d* for t-tests or *f*² for mixed models (Cohen 1988). Statistical significance level was set at *p* <.05 (two-tailed). False discovery rate (FDR) was applied to account for multiple comparisons to limit type I error inflation (Benjamini and Hochberg 1995).

## Results

As reported previously, demographic characteristics were comparable between control participants and those with NMPOU (see Table 1) (Kroll et al. 2018). Consistent with prior findings in indidivuals with opioid dependence, NMPOU participants exhibited higher depression scores (BDI) compared to the control group (Ersche et al. 2006). Opioid craving was generally mild (median = 3, range = 1–8), with no severe withdrawal symptoms during the testing session (see Table 1). Among NMPOU participants, 76% met criteria for moderate to severe OUD according to DSM-5.

**Table 1:** Demographic data and drug use (means and standard deviations)

	Controls	NMPOU	Value	df	*p*
	(*n* = 29)	(*n* = 21)			
Female/male	10/19	5/16	X^2^ = 0.66	1	0.416
Age	26.6 (8.1)	28.7 (10.4)	t=−0.83	48	0.411
BMI	22.2 (2.6)	24.1 (3.5)	t=−1.95	48	0.058
Years of education	11.5 (1.5)	11.1 (2.0)	t = 0.67	35.6	0.507
Verbal IQ	105.2 (11.3)	106.2 (11.7)	t=−0.30	48	0.763
BDI sum score	3.0 (3.3)	9.8 (7.9)	t=−4.16	48	**< 0.001**
Smoking yes/no	18/11	15/6	X^2^ = 0.48	1	0.490
Cigarettes per week^a^	45.4 (35.7)	87.8 (57.7)	t=−2.5	22.5	**0.021**
FTND^a^	1.28 (1.6)	2.87 (2.4)	t=−2.22	23.9	**0.036**
Alcohol yes/no	27/2	19/2	X^2^ = 0.11	1	0.735
Alcohol use in g/week^a^	69.7 (65.5)	51.3 (62.2)	t = 1.03	50	0.309
Cannabis use yes/no	9/20	13/8	X^2^ = 4.71	1	**0.030**
Cannabis use in g/week^a^	0.21 (0.5)	0.62 (0.7)	U = 25.0		**0.025**
**Opioid use**					
Times per week	-	3.9 (3.1)			
Reported ME use in mg/week	-	586.2 (1001)			
Years of use^b^	-	2.9 (0.5–28.0)			
Last opioid use in hours^b^	-	24.0 (1–729.6)			
Craving (NRS)	-	3.3 (2.6)			
Opioid withdrawal (OOWS)	-	1.0 (2.1)			
Positive urine tests for opioids yes/no	0/29	10/11			
ME hair concentration in pg/mg	1 (4)	5084 (7314)			

T-test and Mann-Whitney U test for quantitative data, Chi^2^ for frequency data. Significant *p*-values (*p* <.05) are shown in bold

*BDI* Becks Depression Inventory, *BMI* Body Mass Index, *FTND *Fagerström Test for Nicotine Dependence, *IQ* intelligence quotient, *ME* morphine equivalent, *NRS* Numeric rating scale (1–10), *OOWS* objective opioid withdrawal scale (0–12), *OUD* Opioid Use Disorder

^a^Only within users; ^b^Median (range) are reported

### Stress response of AEA and 2-AG

**Fig. 1:**
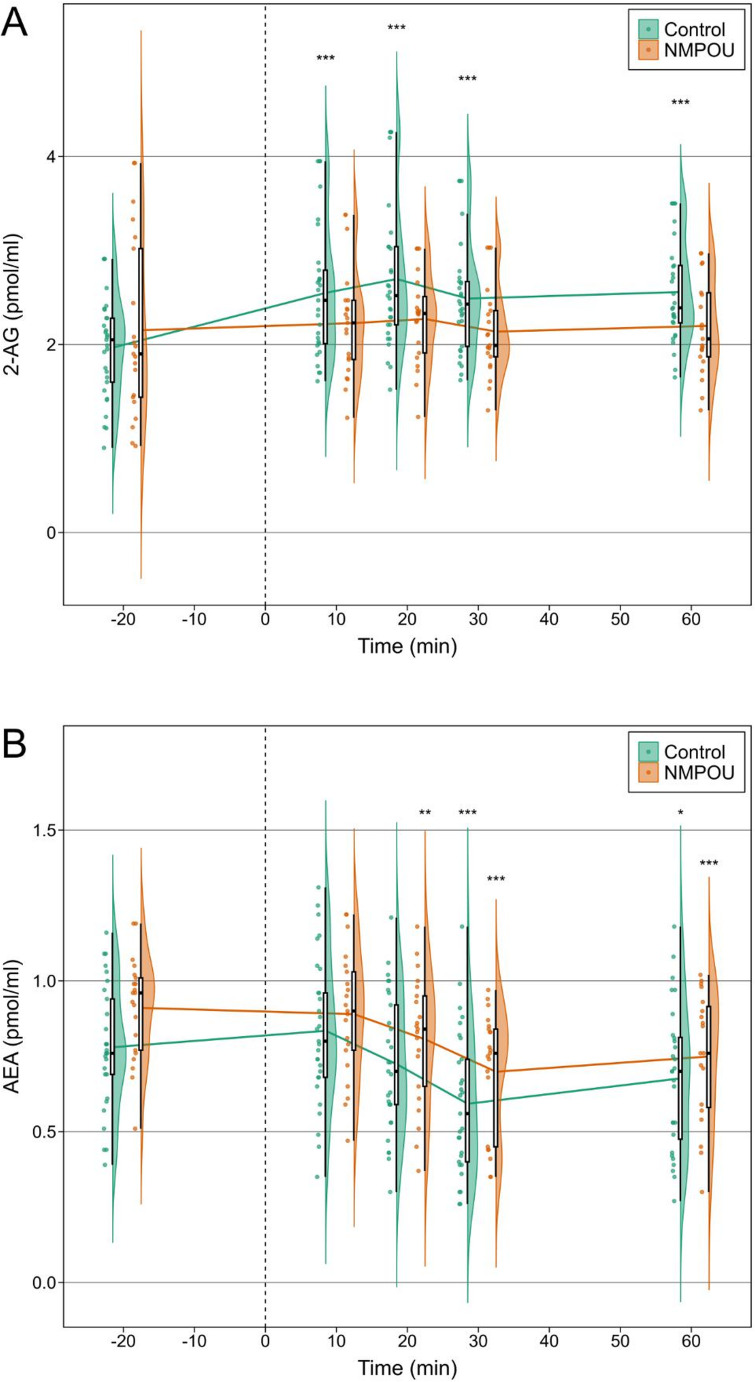
Timeline plots with individual data points showing mean plasma concentrations of (**A**) 2-AG and (**B**) AEA at baseline and 10, 20, 30, and 60 min following the Cyberball task in individuals with NMPOU and healthy controls. Lines indicate group means with half-violin plots showing group distribution and boxplots including median and interquartile range (IQR), with whiskers extending to the most extreme value within 1.5×IQR of the lower and upper quartiles at each timepoint. The dotted line indicates the start of the Cyberball task. Post-hoc dependent t-tests, FDR-corrected, for plasma concentrations at each timepoint compared to T1 within each group, with significant differences denoted by *p <.05, **p <.01, ***p <.001. 2-AG: 2-arachidonoylglycerol, AEA: anandamide

As shown in Fig. 1A, LMMs for repeated measures with 2-AG as the dependent variable yielded no significant main effect of *GROUP* (F(1,102.3) = 0.46, *p* =.501, *f*^2^ = 0.004), but a significant main effect of *TIME* (F(1,48.3) = 11.55, *p* =.001, *f*^2^ = 0.239) and a significant GROUP*TIME interaction (F(1,48.6) = 5.92, *p* =.019, *f*^2^ = 0.122). Post-hoc dependent t-tests demonstrated that this interaction effect was particularly driven by the control group. Within the control group a significant increase of 2-AG levels was found at 10 (t(28) = 4.78, *p* <.001, *d* = 0.89), 20 (t(28) = 5.92, *p* <.001, *d* = 1.10), 30 (t(28) = 4.87, *p* <.001, *d* = 0.91), and 60 min (t(27) = 5.27, *p* <.001, *d* = 1.00) after stress onset compared to baseline (see Fig. 1A), whereas no significant changes were observed in the NMPOU group (*p*-values > 0.326). These effects remained robust after FDR correction (see Supplementary Table S1A & Table S1B). Additional post-hoc independent t-tests at each time point showed significant group differences at 20 (t(48) = 2.23, *p* =.031, *d* = 0.64), 30 (t(48) = 2.23, *p* =.031, *d* = 0.64), and 60 min (t(45) = 2.04, *p* =.047, *d* = 0.61) after stress onset with moderate effect sizes. However, only trend-level significance group differences remained after FDR correction (*p*-values > 0.077, see Supplementary Table S2A).

LMMs for repeated measures with AEA as the dependent variable showed a trend-level significant main effect of *GROUP* (F(1,93.7) = 3.65, *p* =.059, *f*^2^ = 0.039) and *TIME* (F(1,52.0) = 3.05, *p* =.087, *f*^2^ = 0.059), but no GROUP*TIME interaction effects (F(1,52.2) = 0.33, *p* =.566, *f*^2^ = 0.006). Post-hoc dependent t-tests showed significant decreases in AEA levels in both NMPOU (20, 30, and 60 min, *p*-values < 0.004) and controls (30 and 60 min, *p*-values < 0.015) compared to baseline (see Fig. 1B), which remained robust after FDR correction (see Supplementary Table S1C & Table S1D). Post-hoc independent t-tests at each time point showed a significant group difference at baseline (t(48)=−2.38, *p* =.022, *d* = 0.68) but not at subsequent time points (*p*-values > 0.104, see Supplementary Table S2B).

To control for the influence of cannabis use, we conducted additional LMMs including *CANNABIS USE* as a covariate. These analyses did not alter the main findings for 2-AG (see Supplementary Table S3A). The trend-level significance main effects of *GROUP* and *TIME* for AEA remained (*p*-values > 0.075, see Supplementary Table S3B). As the significant GROUP*TIME interaction was only found for 2-AG, we further tested whether this effect was influenced by additional covariates. Adding *BDI*, *BMI*, *SMOKING*, or *ALCOHOL USE* separately, alongside *AGE* and *SEX*, did not change the significance of the GROUP*TIME interaction for 2-AG (see Supplementary Tables S3C–F).

Independent t-tests with endocannabinoid AUC_i_ yielded a significant group difference for 2-AG (t(48) = 2.864, *p* =.006, *d* = 0.82) but not for AEA (t(48) = 1.006, *p* =.320, *d* = 0.29). The control group showed a larger overall increase of 2-AG levels with moderate effect size compared to the NMPOU group. Additional ANCOVAs controlling for *SEX*, *AGE*, and *CANNABIS USE* showed significantly elevated 2-AG concentrations in controls (F(1,45) = 5.89, *p* =.019, *f*^2^ = 0.131) compared to the NMPOU group. No differences were found for AEA (F(1,45) = 0.65, *p* =.426, *f*^2^ = 0.014). Detailed results of ANCOVAs are provided in the Supplementary Table S4A and S4B. Multiple linear regression analysis including *BDI*, *BMI*, *SMOKING*, and *ALCOHOL USE* as predictors indicated that none of these variables significantly predicted 2-AG AUC_i_ (*p*-values > 0.108, see Supplementary Table S5).

To formally test whether the attenuated 2-AG stress response was related to opioid use, we performed Spearman’s rank correlation analyses within the NMPOU group. No significant correlation was found between 2-AG AUCi and opioid use variables (*p-*values *>* 0.107, see Supplementary Fig. S1).

Our exploratory analyses of NAEs and AA revealed no significant GROUP*TIME interaction effects and only weak *GROUP* effects were found for OEA and PEA. Additional analyses controlling for *SEX*,* AGE*, and *CANNABIS USE* did not change the findings. A detailed description of our exploratory analyses is presented in Supplementary Results S1.

### Stress response variables and 2-AG

**Fig. 2:**
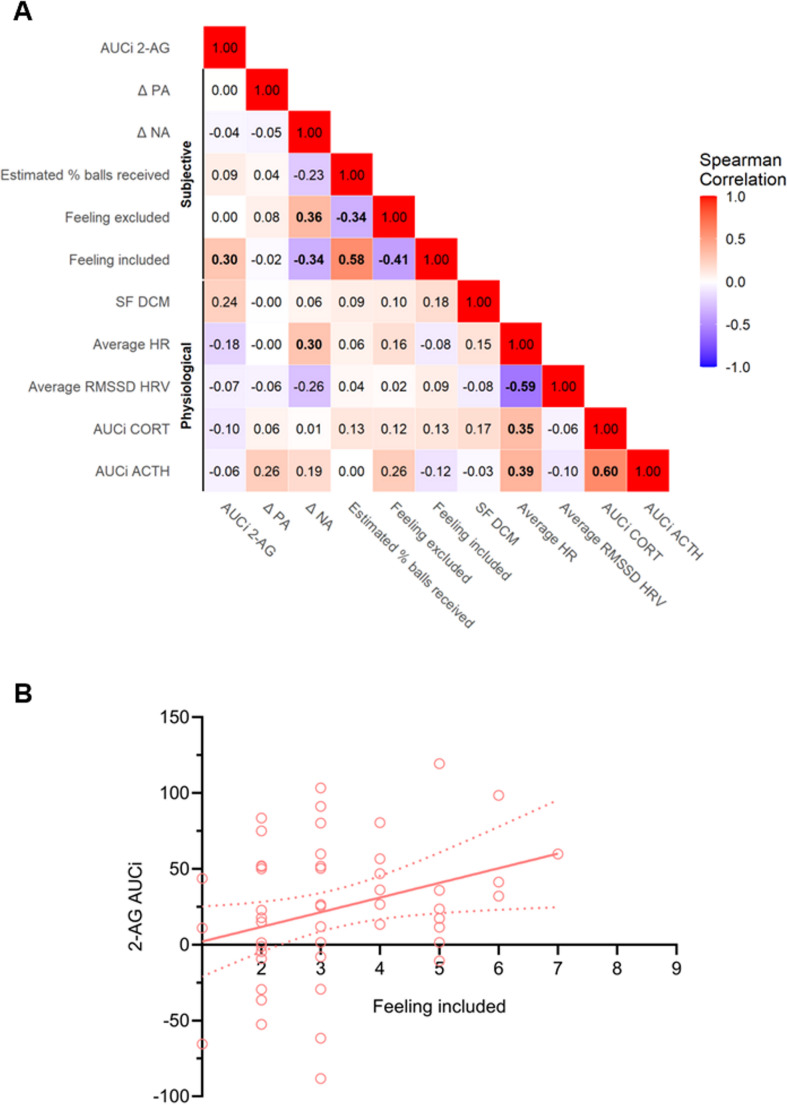
Spearman’s rank correlation over all subjects (*n* = 50) between AUC_i_ 2-AG, subjective social exclusion variables, and physiological stress variables. **A**: Heat matrix of correlation coefficients with significant correlations shown in bold (*p* <.05). **B**: Significant positive correlation between 2-AG AUC_i_ and *FEELING INCLUDED* (r(50) = 0.30, *p* =.035). In red a linear regression is shown (y = 9.664*x − 7.607). ACTH: adrenocorticotropic hormone, AUC_i_: area under the curve with respect to increase, CORT: cortisol, DCM: dynamic casual modeling, HR: heart rate, HRV: heart rate variability, NA: PANAS negative affect, PA: PANAS positive affect, RMSSD: root mean square of successive differences, SF: spontaneous fluctuation, 2-AG: 2-arachidonoylglycerol

Spearman’s rank correlations between AUC_i_ 2-AG and subjective as well as physiological stress markers over all subjects are shown in Fig. 2A. A significant positive association between 2-AG and *FEELING INCLUDED* (r_s_(50) = 0.30, *p* =.035, see Fig. 2B) was found. Additional bootstrapped correlation analysis with 1000 resamples further supported the association between 2-AG AUC_i_ and *FEELING INCLUDED* (r(50) = 0.30, *p* =.035), with a 95% confidence interval of [0.01, 0.51]. A marginal positive association on a trend level was observed between 2-AG and *SPONTANEOUS FLUCTUATIONS DCM* (r_s_(50) = 0.24, *p* =.089). No significant correlations within groups were found (see Supplementary Fig. S3A and Fig. S3B).

## Discussion

Present findings highlight a dysregulated response to experimentally induced social exclusion of the ECS in individuals with chronic NMPOU. In line with preclinical and limited human evidence, we found a distinct increase in peripheral 2-AG levels after a mild stress induction in our healthy control group. In contrast, individuals with NMPOU showed no changes in 2-AG levels after social exclusion indicating a blunted stress response of the ECS. Interestingly, we found a positive correlation between increased 2-AG levels and feeling included after social exclusion suggesting that a stronger response in 2-AG is associated with less subjective stress responses. These findings support animal models proposing that stress-induced increases of 2-AG contribute to the termination of stress response (Evanson et al. 2010; Hill et al. 2011; Ragozzino et al. 2020). Furthermore, the absence of 2-AG reactivity to social stress in individuals with NMPOU may suggests prolonged stress experiences in individuals with chronic opioid use supporting previous human studies reporting dysfunctional psychophysiological stress responses in this population (Kroll et al. 2019).

Animal models have shown a prototypical endocannabinoid response to acute stress, including a rapid decrease in AEA (Gray et al. 2015; Natividad et al. 2017) followed by a delayed increase of 2-AG (Hill et al. 2011; Ragozzino et al. 2020). Our findings of increased 2-AG levels in healthy controls are in line with this preclinical evidence, while we did not detect the expected early decrease in AEA levels. To date, few human studies have investigated stress-related changes in endocannabinoids, reporting heterogeneous results. This variability across studies may result from differences in used stress paradigms, biosampling protocols, and participant characteristics. More precisely, previous studies have shown that pre-analytical sample handling such as the duration of whole blood storage prior to centrifugation – even when stored in ice water or in a fridge – might impact 2-AG plasma levels (Gurke et al. 2019; Kratz et al. 2022; Sens et al. 2023). In our protocol, whole blood samples taken at the beginning of the study were kept in ice cold water for a longer period before centrifugation than samples taken at later time points. This might have affected 2-AG plasma levels, which may have led to an increase in 2-AG levels over time. However, since we did not find a linear increase in 2-AG over time in both groups, our results cannot be explained by pre-analytical handling per se. Instead, despite identical sample handling, we found a 2-AG plasma peak only in the control group, indicating a significant group difference in 2-AG stress response and supporting our assumption of a dysfunctional stress response in individuals with NMPOU.

The most common stress paradigms used in laboratory settings are the Maastrich Acute Stress Task (MAST; (Smeets et al. 2012), which has stronger physiological and moderate psychosocial components, and the Trier Social Stress Test (TSST; (Kirschbaum et al. 1993), which is more focused around the psychosocial stress component. Notably, both the TSST and MAST elicit robust responses in subjective stress ratings, the autonomic nervous system, and in cortisol levels, reflecting reliable HPA axis reactivity (Smeets et al. 2012; Allen et al. 2016; Sequeira et al. 2021). Studies implementing the MAST found decreased AEA levels at different time points post-stressor but no significant 2-AG changes (Mayo et al. 2018, 2020), whereas Ney and colleagues (2021) reported an increase in salivary 2-AG approximately 15 min after stress onset, but reported no significant changes in AEA or plasma endocannabinoid concentrations. In contrast, studies using the TSST reported increased 2-AG levels approximately 17 min after stress onset (Hill et al. 2009b), elevations in both AEA and 2-AG approximately 10 min following stress onset (Crombie et al. 2019), or only increased AEA approximately 10 min after stress onset but no 2-AG changes (Dlugos et al. 2012). Additionally, a parabolic flight study found increased 2-AG levels post-stressor in individuals without motion sickness compared to individuals who developed motion sickness, with a negative correlation between 2-AG and salivary cortisol in the subpopulation without motion sickness (Choukèr et al. 2010). In contrast, the Cyberball task has been primarily associated with strong feelings of subjective distress and negative affect (Hartgerink et al. 2015), whereas cardiovascular reactivity (e.g., increased HR (Iffland et al. 2014); increased blood pressure (Eres et al. 2021) and cortisol stress responses typically showed moderate to small or even absent effects in healthy subjects (Gaffey and Wirth 2014; Radke et al. 2018; Kroll et al. 2019). While we used a more self-related and mild stressor of social exclusion, our results are broadly consistent with previous research implementing the TSST as a psychosocial stressor (Hill et al. 2009b; Crombie et al. 2019). Notably, most human studies have assessed 2-AG either before or after 20 min following stress onset, with Hill and colleagues (2009b) being the only study to report increased plasma 2-AG levels around this time, closely aligning with our observed peak at 20 min after stress onset.

Of note, we previously showed hyperactivity of the HPA axis in the same NMPOU samples compared to healthy controls (Kroll et al. 2019). This is particularly interesting given that the Cyberball task as a mild stressor usually does not elicit a strong HPA axis reactivity in healthy subjects (Seidel et al. 2013; Gaffey and Wirth 2014) in contrast to the MAST (Smeets et al. 2012) or TSST (Kirschbaum et al. 1993). Together with our present results, findings indicate a dysfunctional stress response in chronic opioid users at both levels, the HPA axis and the ECS. Notably, we were not able to find correlations between cortisol, ACTH, and endocannabinoid plasma levels. This might be explained by differences in the time course of circulating glucocorticoid and endocannabinoid levels in the peripheral and central nervous systems or, alternatively, by individual differences in the responsiveness of both stress systems. In humans, 2-AG synthesis may be more strongly driven by SNS activation than by glucocorticoid signaling, as observed in rodent models (Hill et al. 2009b). Stress-induced SNS activation rapidly increases catecholamine release, which in turn bind to α-adrenergic receptors among other targets (Charmandari et al. 2005). This receptor activation stimulates phospholipase C activity (Zemkova et al. 2011), a key enzyme in generating diacylglycerols, the primary precursors of 2-AG (Bisogno 2008). Since CB1 receptors are located on sympathetic nerve terminals, their activation by endocannabinoids plays a crucial role in modulating norepinephrine release, thereby modulating sympathetic activity (Ishac et al. 1996; Pfitzer et al. 2005; Tam et al. 2008). This suggests that SNS-driven 2-AG synthesis may serve as a rapid regulatory mechanism to modulate stress responses, particularly by counteracting excessive sympathetic outflow. Although we found only a weak correlation between SF skin conductance measure as a proxy for SNS activity and 2-AG AUC_i_, our results may show preliminary evidence for a relationship between SNS-induced 2-AG reactivity to social stress, which needs to be tested in future studies in more detail.

Our findings indicate that a stronger 2-AG stress response is associated with greater feelings of inclusion after social exclusion, supporting the role of 2-AG as a potential factor to mediate stress-resilience (Patel et al. 2005; Hill et al. 2010). Accordingly, inhibition of monoacylglycerol lipase (MAGL), the enzyme responsible for degrading 2-AG and increasing 2-AG levels, has been shown to produce anxiolytic effects in mice exposed to aversive environments, effects which were reversed by co-administration of a CB1 receptor inverse agonist (Sciolino et al. 2011). Furthermore, inhibition of MAGL in stress-susceptible mice increased their resilience, whereas 2-AG depletion led to anxiety-like behavior following stress exposure (Bluett et al. 2017). Our present findings of blunted 2-AG stress response in individuals with chronic NMPOU might indicate that individuals with OUD are more susceptible to stress in terms of a prolonged subjective stress reaction. An inadequate termination of stress response may further result in increased opioid craving strengthening the vicious cycle of opioid addiction. Since we did not find associations between endocannabinoid levels and opioid use variables, results suggest that blunted 2-AG response might be rather related to chronic opioid use itself than to dose-dependent effects. Therefore, targeting 2-AG might be a potential pharmacological target of OUD treatment. The dual inhibition of both MAGL and FAAH has shown promise in preclinical models by reducing opioid withdrawal symptoms and drug-seeking behaviors (Ramesh et al. 2013; Wilkerson et al. 2016). Furthermore, a recent preclinical study demonstrated that inhibition of MAGL, but not FAAH, reduced the rewarding properties of opioids while preserving their analgesic efficacy (Martínez-Rivera et al. 2024). Nevertheless, preclinical findings should be interpreted with caution, as methodologies in animal models have varied significantly, and the relevance of these interactions to human populations remains poorly understood (Aghaei et al. 2023). Moreover, clinical studies targeting the ECS with exogenous cannabinoids for OUD treatment showed modest results (Babalonis and Walsh 2020; Ganesh et al. 2024). Continued investigation into the bidirectional relationship between the ECS and the opioid system could provide critical insights for developing novel treatments for OUD. To date, only a single MAGL inhibitor has reached phase II clinical trials, where it was evaluated for reducing tics in Tourette syndrome. Although the treatment was generally safe, the trial was discontinued after failing to meet its primary endpoint (Müller-Vahl et al. 2021). Currently, new selective and reversible MAGL inhibitors are being developed, which could reduce the pharmacological side effects and present a novel approach in targeting the ECS (Yu et al. 2024).

In line with our previous study results, we found significant group differences in NAE levels, particularly for OEA and PEA, throughout all time points. AEA levels were significantly higher at baseline in the NMPOU group compared to controls, and decreased over time in both groups. No significant stress-induced changes over time were found for NAEs and AA. Particularly the absence of an early AEA stress response in our healthy control sample is surprising, as we would have expected a change in AEA concentrations based on animal findings reporting a rapid reduction in AEA levels across limbic brain regions following acute stress exposure (Patel et al. 2005; Hill et al. 2009a; Gray et al. 2015). However, human studies so far reported varying AEA responses to stress across different stressor types and timepoints. A reduction of AEA after the MAST, a more physiological stressor, was found approximately 10 min after stress onset (Mayo et al. 2018), approximately 30 min after stress onset (Mayo et al. 2020), or not at all (Ney et al. 2021). In contrast, studies using the TSST, a more psychosocial stressor, found no changes in AEA levels approximately 17 min and 47 min after stress onset (Hill et al. 2009b), while others reported an increase in AEA approximately 10 min after stress onset (Dlugos et al. 2012; Crombie et al. 2019), further underlining possible differences in the ECB stress response based on the nature of the stressor. While we used a rather mild psychosocial stressor with a short duration of stress induction compared to the MAST and TSST, it is possible that the timing of our first blood draw post-stressor was too late to capture immediate stress-induced changes in AEA. Given that our sampling began 10 min after the onset of the Cyberball task, we may have missed the rapid fluctuations afters stress induction in AEA found both in animal models and previous human studies using more immediate post-stressor collection after a longer stress paradigm (Dlugos et al. 2012; Mayo et al. 2018; Crombie et al. 2019).

Although we did not find a significant rapid drop in AEA levels immediately after the onset of stress, we indeed found a decrease in AEA levels over time, which seems to be stable 30 to 60 min after stress induction. Therefore, our data might suggest a delayed AEA stress response, which occurred even after 2-AG response. However, preclinical findings are rarely translatable to humans, and the prototypical AEA/2-AG stress response reported in animal models of stress might occur over a different time period in humans, particularly with regard to peripheral endocannabinoid levels. Another possible explanation for the decrease in AEA levels over time might be diurnal fluctuations, which has been reported previously (Murillo-Rodriguez et al. 2006; Hanlon 2020). However, since AEA levels did not decrease continuously at the latest time point between 30 and 60 min, this explanation is not robustly supported by our data and more time points are needed to test a potential diurnal decrease. Furthermore, pre-analytical sample handling may also account for our AEA findings. Earlier samples underwent longer pre-centrifugation delays of whole blood stored in ice cold water. It has been reported that AEA increases in these conditions, which could lead to a potential decrease of AEA over time (Vogeser et al. 2006; Gurke et al. 2019; Sens et al. 2023). However, since all samples were handled identically, this represents a systematic bias that does not affect group-level findings. Moreover, since we did not find a linear decrease of AEA after 30 and 60 min, pre-analytical handling may not explain our AEA results per se. To determine which explanation might be the most likely interpretation of our AEA findings, future studies should address the reported issues by optimizing the sampling schedule and pre-analytical handling.

The present study has several considerations to note: (i) The cross-sectional design precludes definitive conclusions about causal relationships between chronic opioid use and ECS dysregulation, although it provides valuable initial insights into their association. (ii) The sample size of the NMPOU group, while small, may have limited the statistical power to detect more nuanced effects or interaction terms. However, this was partially mitigated by the use of bootstrapping techniques, which enhanced the robustness of our statistical analyses. Furthermore, the moderate effect sizes observed in our analyses suggest meaningful associations that warrant further exploration. (iii) While plasma endocannabinoid levels, as lipid molecules capable of readily crossing the blood-brain barrier, serve as a useful and accessible proxy for systemic dynamics (Banks et al. 1997; Hillard 2017), they may not fully capture ECS activity within the central nervous system (Meier et al., 2024), where stress responses are predominantly regulated. Nonetheless, translational research suggests that systemic endocannabinoid concentrations that are influenced by genetic variations such as FAAH polymorphisms are correlated with central ECS activity in both animals and humans (Mayo et al. 2018), but see also (Koethe et al. 2009).

In sum, this study supports previous findings of dysfunctional stress response in OUD also at the level of the ECS. Particularly blunted 2-AG stress response in individuals with chronic NMPOU might indicate impaired termination of stress reactivity suggesting prolonged stress experience. Together with our previous findings of elevated basal AEA levels in the NMPOU group associated with less feelings of social exclusion, present results suggest that the ECS in terms of tonic AEA levels and phasic 2-AG reactivity plays a critical role in stress response in individuals with chronic opioid use by affecting subjective stress experiences as well as termination of stress response, respectively. Given that stress plays a crucial role in the vicious cycle of substance use disorders, including OUD, an in-depth understanding of the neurobiological mechanisms underpinning altered stress response in individuals with chronic opioid use could lead to novel and improved treatment options for OUD. Preclinical studies exploring the therapeutic potential of ECS manipulation in OUD are promising (Karimi-Haghighi et al. 2024). These advancements highlight the potential of targeting the ECS as a promising avenue for therapeutic intervention in OUD, underscoring the need for continued translational research to refine and optimize these strategies for clinical application.

## Supplementary Information

Below is the link to the electronic supplementary material.

Supplementary Material 1 (DOCX 1.87 MB)

## Data Availability

The data that support the findings of this study are not openly available due to reasons of sensitivity and are available from the corresponding author upon reasonable request.
