# Supplementary material for: Endocannabinoid response to social stress in chronic non-medical prescription opioid users
Source: Psychopharmacology (Berl). 2025 Nov 5;243(2):427–41. doi: 10.1007/s00213-025-06950-4 (PMC12904892; doi:10.1007/s00213-025-06950-4)
Supplement: Supplementary file 1 — Supplementary Material 1 (DOCX 1.87 MB) [file 213_2025_6950_MOESM1_ESM.docx]

Supplementary Materials

Endocannabinoid Response to Social Stress in Chronic Non-Medical Prescription Opioid Users

Vinzenz K. Schmid, Boris B. Quednow, Philip Meier, Jürg Gertsch, Sara L. Kroll

**Method S1** Quantification of endocannabinoids and associated lipids in plasma samples of NMPOU and control participants

For the quantification of endocannabinoids and associated lipids in plasma samples, an earlier published method was applied using a liquid-liquid extraction followed by liquid chromatography-electrospray ionization-tandem mass spectrometry (LC-ESI-MS/MS) analysis [1]. In brief, of each participant 200 µL of plasma were added to 0.75 mL of ethyl acetate:hexane (9:1, HPLC grade, VWR Chemicals, Poland) with 0.1 % formic acid (Sigma-Aldrich Chemie GmbH, Germany) already containing 1 µL of an internal standard mixture prepared in absolute ethanol (VWR International, France). After addition of 50 µL 0.1 M formic acid, the samples were strongly vortexed and sonicated for five minutes in a pre-cooled water bath followed by a centrifugation for 10 minutes with 1620 g at 4°C. The samples were located on dry ice for 10 minutes to freeze the lower aqueous phase and the upper organic phase was collected and evaporated in plastic tubes for 45 min using an Eppendorf Concentrator plus (Eppendorf AG, Germany). The dried pellets were reconstituted in 35 µL of Acetonitrile:H2O (8:2, HPLC grade, Fisher Scientific, UK) and 10 µL of the resuspended samples were injected into the LC-ESI-MS/MS system (linear ion trap quadrupole LC-MS/MS mass spectrometer, QTRAP 5500, AB Sciex Instruments, Massachusetts, USA). The LC-ESI-MS/MS conditions were used as described before [1] and the analytes 2-arachidonoylglycerol (2-AG), anandamide (AEA), linoleoyl ethanolamide (LEA), oleoylethanolamide (OEA), palmitoylethanolamide (PEA), and stearoyl ethanolamide (SEA) were analyzed using the turbo-ion spray interface operated in positive mode, while arachidonic acid (AA) was analyzed with the turbo-ion spray interface operated in negative mode. The used MRM transitions and internal standards for each analyte are summarized in the supplementary table below. For the quantification a calibration curve was used with increasing amounts of analyte standards in a matrix of 1 % bovine serum albumin (fatty acid free, Sigma-Aldrich, Missouri, USA) in ddH2O. The processing and the measurements of the calibration samples was performed in the exact same way as the plasma samples. A linear calibration curve for each analyte with the quantified area ratio (analyte peak area/internal standard peak area) and the known analyte concentration was generated with the software Analyst 1.6.3 (AB Sciex, Massachusetts, USA) and the concentration of each analyte was calculated for the sample preparations. To determine the analyte concentration in plasma, the quantified amount of each analyte was normalized to the sample volume (200 µL) used for the extraction. 2-AG can isomerize to 1-AG with several different storage and extraction methods [2]. In the chromatography 1-AG is released at a slightly different retention time leading to a second peak in the chromatogram. To analyze the biological levels of 2-AG in the plasma samples the peaks of 2-AG and 1-AG were quantified together.

**Result S1** Exploratory Analysis of NAEs and AA

Exploratory analyses for stress response of NAEs yielded significant *GROUP* effects for OEA (F(1,82.1)=9.05, *p*=.003, *f*^2^=.110) and PEA (F(1,93.3)=9.27, *p*=.003, *f*^2^=.099) but no *TIME* and *GROUP*TIME* interactions. No significant effects for LEA as the dependent variable were found (*p-*values >.090). A significant *TIME* effect (F(1,218.5)=14.0, *p*<.001, *f*^2^=.064) but no *GROUP* effects and *GROUP*TIME* interaction was found for AA. All LMMs, including non-significant findings and mean plasma concentrations before and after the Cyberball task, are presented in Supplementary Figure S2 and Table S6A.

Additional analyses with *CANNABIS USE* did not change the main findings for OEA, PEA, and AA (see supplementary materials Table S6B).

Independent t-tests with NAEs and AA as dependent variables showed a significant group difference for PEA (t(48)=2.58, *p*=.013, *d*=.74), with the control group exhibiting larger AUC_i_ values than the NMPOU group. No significant group differences were found for the remaining NAEs and AA (*p*-values >.168). ANCOVAs with *SEX* and *AGE* did not change the results. Further adding *CANNABIS USE* into the ANCOVA did not change the results. Detailed results for all analyses are provided in the Supplementary Tables S7A-C.

**Table S1A** Paired samples t-tests for 2-AG in controls

|  | **Mean** | **SD** | **t** | ***df*** | ***p*** | ***p_FDR_*** |
| --- | --- | --- | --- | --- | --- | --- |
| Pair 1  (T2 – T1) | 0.582 | 0.657 | 4.78 | 28 | **<0.001** | **<0.001** |
| Pair 2  (T3 – T1) | 0.730 | 0.664 | 5.92 | 28 | **<0.001** | **<0.001** |
| Pair 3  (T4 – T1) | 0.522 | 0.577 | 4.87 | 28 | **<0.001** | **<0.001** |
| Pair 4  (T5 – T1) | 0.588 | 0.590 | 5.27 | 27 | **<0.001** | **<0.001** |

FDR: false discovery rate, 2-AG: 2-arachidonylglycerol

**Table S1B** Paired samples t-tests for 2-AG in NMPOU

|  | **Mean** | **SD** | **t** | ***df*** | ***p*** | ***p_FDR_*** |
| --- | --- | --- | --- | --- | --- | --- |
| Pair 1  (T2 – T1) | 0.076 | 0.700 | 0.50 | 20 | 0.623 | 0.831 |
| Pair 2  (T3 – T1) | 0.120 | 0.743 | 0.74 | 20 | 0.470 | 0.831 |
| Pair 3  (T4 – T1) | -0.014 | 0.861 | -0.08 | 20 | 0.940 | 0.940 |
| Pair 4  (T5 – T1) | 0.188 | 0.810 | 1.01 | 18 | 0.326 | 0.831 |

FDR: false discovery rate, 2-AG: 2-arachidonylglycerol

**Table S1C** Paired samples t-tests for AEA in controls

|  | **Mean** | **SD** | **t** | ***df*** | ***p*** | ***p_FDR_*** |
| --- | --- | --- | --- | --- | --- | --- |
| Pair 1  (T2 – T1) | 0.054 | 0.213 | 1.38 | 28 | 0.179 | 0.179 |
| Pair 2  (T3 – T1) | -0.056 | 0.199 | -1.52 | 28 | 0.140 | 0.179 |
| Pair 3  (T4 – T1) | -0.188 | 0.215 | -4.70 | 28 | **<** **0.001** | **<0.001** |
| Pair 4  (T5 – T1) | -0.117 | 0.239 | -2.59 | 27 | **0.015** | **0.031** |

FDR: false discovery rate, AEA: anandamide

**Table S1D** Paired samples t-tests for AEA in NMPOU

|  | **Mean** | **SD** | **t** | ***df*** | ***p*** | ***p_FDR_*** |
| --- | --- | --- | --- | --- | --- | --- |
| Pair 1  (T2 – T1) | -0.021 | 0.151 | -0.64 | 20 | 0.533 | 0.532 |
| Pair 2  (T3 – T1) | -0.104 | 0.144 | -3.31 | 20 | **0.004** | **0.005** |
| Pair 3  (T4 – T1) | -0.212 | 0.151 | -6.45 | 20 | **<0.001** | **<0.001** |
| Pair 4  (T5 – T1) | -0.142 | 0.144 | -4.29 | 18 | **<0.001** | **<0.001** |

FDR: false discovery rate, AEA: anandamide

**Table S2A** Independent t-tests for 2-AG

|  | **Controls** | **NMPOU** | **Value** | ***df*** | ***p*** | ***p_FDR_*** |
| --- | --- | --- | --- | --- | --- | --- |
|  | (n=29) | (n=21) |  |  |  |  |
| T1 | 1.97 (0.54) | 2.15 (0.96) | t = -0.80 | 29.2^a^ | 0.432 | 0.432 |
| T2 | 2.55 (0.70) | 2.23 (0.59) | t = 1.71 | 48 | 0.094 | 0.118 |
| T3 | 2.70 (0.77) | 2.27 (0.48) | t = 2.23 | 48 | 0.031 | 0.077 |
| T4 | 2.49 (0.60) | 2.14 (0.48) | t = 2.23 | 48 | 0.031 | 0.077 |
| T5^b^ | 2.55 (0.55) | 2.22 (0.54) | t = 2.04 | 45 | 0.047 | 0.079 |

FDR: false discovery rate, 2-AG: 2-arachidonylglycerol. ^a^Equal variances not assumed; ^b^Three missing plasma samples (Controls, n=28; NMPOU, n=19)

**Table S2B** Independent t-tests for AEA

|  | **Controls** | **NMPOU** | **Value** | ***df*** | ***p*** | ***p_FDR_*** |
| --- | --- | --- | --- | --- | --- | --- |
|  | (n=29) | (n=21) |  |  |  |  |
| T1 | 0.78 (0.21) | 0.91 (0.17) | t = -2.38 | 48 | 0.022 | 0.108 |
| T2 | 0.83 (0.24) | 0.89 (0.21) | t = -0.84 | 48 | 0.406 | 0.406 |
| T3 | 0.72 (0.21) | 0.81 (0.21) | t = -1.29 | 48 | 0.204 | 0.339 |
| T4 | 0.59 (0.24) | 0.70 (0.20) | t = -1.66 | 48 | 0.104 | 0.261 |
| T5^a^ | 0.68 (0.24) | 0.75 (0.22) | t = -1.06 | 45 | 0.296 | 0.370 |

FDR: false discovery rate, AEA: anandamide. ^a^Three missing plasma samples (Controls, n=28; NMPOU, n=19)

**Table S3A** Linear mixed models for repeated measures parameters with the fixed factor *GROUP*, *TIME*, and *GROUP*TIME* interactions controlled for *AGE* and *SEX*

Dependent variable: *2-AG*

|  | Estimate | SE | CI | *df* | t | *p* |
| --- | --- | --- | --- | --- | --- | --- |
| **(Intercept)** | **0.913** | **0.448** | **0.022 – 1.803** | **88.151** | **2.04** | **0.045** |
| GROUP | 0.143 | 0.212 | -0.278 – 0.564 | 102.320 | 0.68 | 0.501 |
| **TIME** | **0.272** | **0.080** | **0.111 – 0.433** | **48.280** | **3.40** | **0.001** |
| **GROUP*TIME** | **-0.130** | **0.053** | **-0.237 – -0.023** | **48.647** | **-2.43** | **0.019** |
| AGE | 0.012 | 0.008 | -0.004 – 0.028 | 52.538 | 1.54 | 0.129 |
| **SEX** | **0.347** | **0.151** | **0.043 – 0.651** | **50.133** | **2.29** | **0.026** |

Dependent variable: *AEA*

|  | Estimate | SE | CI | *df* | t | *p* |
| --- | --- | --- | --- | --- | --- | --- |
| **(Intercept)** | **0.889** | **0.157** | **0.575 – 1.203** | **66.126** | **5.65** | **<.001** |
| GROUP | 0.127 | 0.067 | -0.005 – 0.259 | 93.675 | 1.91 | 0.059 |
| TIME | -0.033 | 0.019 | -0.072 – 0.005 | 51.957 | -1.75 | 0.087 |
| GROUP*TIME | -0.007 | 0.013 | -0.033 – 0.018 | 52.230 | -0.58 | 0.566 |
| AGE | -0.002 | 0.003 | -0.008 – 0.004 | 50.561 | -0.80 | 0.426 |
| SEX | -0.066 | 0.059 | -0.184 – 0.052 | 49.773 | -1.13 | 0.265 |

**Table S3B** Linear mixed models for repeated measures parameters with the fixed factor *GROUP*, *TIME*, and *GROUP*TIME* interactions controlled for *AGE*, *SEX,* and *CANNABIS USE.*

Dependent variable: *2-AG*

|  | Estimate | SE | CI | *df* | t | *p* |
| --- | --- | --- | --- | --- | --- | --- |
| (Intercept) | 0.628 | 0.513 | -0.393 – 1.649 | 78.654 | 1.23 | 0.224 |
| GROUP | 0.202 | 0.218 | -0.230 – 0.634 | 103.774 | 0.93 | 0.355 |
| **TIME** | **0.272** | **0.080** | **0.111 – 0.434** | **47.877** | **3.39** | **0.001** |
| **GROUP*TIME** | **-0.130** | **0.054** | **-0.238 – -0.022** | **48.244** | **-2.43** | **0.019** |
| AGE | 0.009 | 0.008 | -0.008 – 0.025 | 52.197 | 1.08 | 0.287 |
| **SEX** | **0.361** | **0.150** | **0.059 – 0.662** | **50.027** | **2.40** | **0.020** |
| CANNABIS USE | 0.169 | 0.154 | -0.140 – 0.478 | 49.518 | 1.10 | 0.278 |

Dependent variable: *AEA*

|  | Estimate | SE | CI | *df* | t | *p* |
| --- | --- | --- | --- | --- | --- | --- |
| **(Intercept)** | **0.897** | **0.187** | **0.524 – 1.271** | **60.830** | **4.81** | **<.001** |
| GROUP | 0.125 | 0.070 | -0.013 – 0.264 | 89.685 | 1.80 | 0.075 |
| TIME | -0.033 | 0.019 | -0.072 – 0.005 | 51.974 | -1.75 | 0.087 |
| GROUP*TIME | -0.007 | 0.013 | -0.033 – 0.018 | 52.247 | -0.58 | 0.566 |
| AGE | -0.002 | 0.003 | -0.009 – 0.004 | 50.443 | -0.72 | 0.474 |
| SEX | -0.066 | 0.059 | -0.185 – 0.052 | 49.762 | -1.13 | 0.264 |
| CANNABIS USE | -0.005 | 0.060 | -0.126 – 0.116 | 49.597 | -0.08 | 0.933 |

**Table S3C** Linear mixed models for repeated measures parameters with the fixed factor *GROUP*, *TIME*, and *GROUP*TIME* interactions controlled for *AGE*, *SEX,* and *BDI.*

Dependent variable: *2-AG*

|  | Estimate | SE | CI | *df* | t | *p* |
| --- | --- | --- | --- | --- | --- | --- |
| **(Intercept)** | **0.910** | **0.447** | **0.022 – 1.798** | **87.726** | **2.04** | **0.045** |
| GROUP | 0.189 | 0.229 | -0.264 – 0.643 | 103.693 | 0.83 | 0.410 |
| **TIME** | **0.271** | **0.080** | **0.111 – 0.431** | **48.692** | **3.41** | **0.001** |
| **GROUP*TIME** | **-0.129** | **0.053** | **-0.236 – -0.023** | **49.057** | **-2.44** | **0.019** |
| AGE | 0.012 | 0.008 | -0.003 – 0.028 | 52.615 | 1.59 | 0.117 |
| **SEX** | **0.327** | **0.155** | **0.017 – 0.638** | **50.249** | **2.12** | **0.039** |
| BDI | -0.007 | 0.013 | -0.032 – 0.018 | 50.026 | -0.55 | 0.587 |

**Table S3D** Linear mixed models for repeated measures parameters with the fixed factor *GROUP*, *TIME*, and *GROUP*TIME* interactions controlled for *AGE*, *SEX,* and *BMI.*

Dependent variable: *2-AG*

|  | Estimate | SE | CI | *df* | t | *p* |
| --- | --- | --- | --- | --- | --- | --- |
| (Intercept) | 0.127 | 0.574 | -1.017 – 1.270 | 72.340 | 0.22 | 0.826 |
| GROUP | 0.082 | 0.211 | -0.338 – 0.501 | 102.405 | 0.39 | 0.700 |
| **TIME** | **0.272** | **0.081** | **0.110 – 0.434** | **48.168** | **3.38** | **0.001** |
| **GROUP*TIME** | **-0.130** | **0.054** | **-0.238 – -0.022** | **48.539** | **-2.42** | **0.019** |
| AGE | 0.005 | 0.008 | -0.012 – 0.021 | 51.959 | 0.54 | 0.591 |
| SEX | 0.261 | 0.151 | -0.042 – 0.564 | 50.256 | 1.73 | 0.090 |
| **BMI** | **0.053** | **0.025** | **0.002 – 0.104** | **49.665** | **2.09** | **0.042** |

**Table S3E** Linear mixed models for repeated measures parameters with the fixed factor *GROUP*, *TIME*, and *GROUP*TIME* interactions controlled for *AGE*, *SEX,* and *SMOKING.*

Dependent variable: *2-AG*

|  | Estimate | SE | CI | df | t | *p* |
| --- | --- | --- | --- | --- | --- | --- |
| (Intercept) | 0.664 | 0.456 | -0.242 – 1.571 | 88.123 | 1.46 | 0.149 |
| GROUP | 0.186 | 0.211 | -0.233 – 0.604 | 101.718 | 0.88 | 0.381 |
| **TIME** | **0.272** | **0.081** | **0.110 – 0.434** | **47.650** | **3.37** | **0.001** |
| **GROUP*TIME** | **-0.129** | **0.054** | **-0.238 – -0.021** | **48.014** | **-2.40** | **0.020** |
| AGE | 0.008 | 0.008 | -0.008 – 0.023 | 52.333 | 0.95 | 0.347 |
| **SEX** | **0.306** | **0.148** | **0.009 – 0.603** | **50.197** | **2.07** | **0.044** |
| SMOKING | 0.282 | 0.149 | -0.017 – 0.581 | 50.385 | 1.89 | 0.064 |

**Table S3F** Linear mixed models for repeated measures parameters with the fixed factor *GROUP*, *TIME*, and *GROUP*TIME* interactions controlled for *AGE*, *SEX,* and *ALCOHOL USE.*

Dependent variable: *2-AG*

|  | Estimate | SE | CI | *df* | t | *p* |
| --- | --- | --- | --- | --- | --- | --- |
| (Intercept) | 0.895 | 0.493 | -0.086 – 1.877 | 82.351 | 1.82 | 0.073 |
| GROUP | 0.143 | 0.212 | -0.278 – 0.564 | 102.142 | 0.68 | 0.501 |
| **TIME** | **0.272** | **0.080** | **0.111 – 0.433** | **48.173** | **3.40** | **0.001** |
| **GROUP*TIME** | **-0.130** | **0.053** | **-0.237 – -0.023** | **48.541** | **-2.44** | **0.019** |
| AGE | 0.012 | 0.008 | -0.005 – 0.028 | 51.797 | 1.44 | 0.157 |
| **SEX** | **0.346** | **0.152** | **0.042 – 0.650** | **50.050** | **2.28** | **0.027** |
| ALCOHOL USE | 0.023 | 0.269 | -0.516 – 0.562 | 51.036 | 0.09 | 0.931 |

**Table S4A** ANCOVAs with the dependent variables AUC_i_ 2-AG and AEA controlled for *SEX* and *AGE*

|  | **Controls** | **NMPOU** | ***F*** | ***df1*** | ***df2*** | ***p*** |
| --- | --- | --- | --- | --- | --- | --- |
|  | (n=29) | (n=21) |  |  |  |  |
| **2-AG** | **38.26 (36.36)** | **3.14 (50.44)** | **7.23** | **1** | **45** | **0.010** |
| AEA | -4.86 (13.05) | -8.18 (8.90) | 0.78 | 1 | 45 | 0.381 |

**Table S4B** ANCOVAs with the dependent variables AUC_i_ 2-AG and AEA controlled for *SEX*, *AGE* and *CANNABIS USE*

|  | **Controls** | **NMPOU** | ***F*** | ***df1*** | ***df2*** | ***p*** |
| --- | --- | --- | --- | --- | --- | --- |
|  | (n=29) | (n=21) |  |  |  |  |
| **2-AG** | **38.3 (36.4)** | **3.1 (50.4)** | **5.89** | **1** | **45** | **0.019** |
| AEA | -4.86 (13.05) | -8.18 (8.90) | 0.65 | 1 | 45 | 0.426 |

**Table S5** Multiple linear regression model with the dependent variable AUC_i_ 2-AG and independent variables *BDI, BMI, SMOKING, and ALCOHOL USE*

| Predictor | B | SE | *β* | t | *p* |
| --- | --- | --- | --- | --- | --- |
| (Constant) | 99.493 | 47.511 |  | 1.99 | 0.053 |
| BDI | -1.402 | 0.897 | -0.213 | -1.56 | 0.125 |
| BMI | 3.188 | 1.944 | 0.228 | 1.64 | 0.108 |
| SMOKING | 21.208 | 12.946 | 0.234 | 1.64 | 0.108 |
| ALCOHOL USE | -1.258 | 22.203 | -0.008 | -0.06 | 0.955 |

**Table S6A** Linear mixed models for repeated measures parameters with the fixed factor *GROUP*, *TIME*, and *GROUP*TIME* interactions controlled for *AGE* and *SEX*

Dependent variable: *OEA*

|  | Estimate | SE | CI | *df* | t | *p* |
| --- | --- | --- | --- | --- | --- | --- |
| **(Intercept)** | **1.726** | **0.370** | **0.995 – 2.458** | **62.684** | **4.72** | **<.001** |
| **GROUP** | **0.457** | **0.152** | **0.155 – 0.759** | **82.126** | **3.01** | **0.003** |
| TIME | -0.047 | 0.041 | -0.130 – 0.035 | 35.892 | -1.17 | 0.251 |
| GROUP*TIME | -0.030 | 0.027 | -0.085 – 0.025 | 36.116 | -1.12 | 0.272 |
| AGE | -0.006 | 0.007 | -0.020 – 0.008 | 50.640 | -0.82 | 0.418 |
| SEX | -0.220 | 0.138 | -0.497 – 0.058 | 49.970 | -1.59 | 0.118 |

Dependent variable: *PEA*

|  | Estimate | SE | CI | *df* | t | *p* |
| --- | --- | --- | --- | --- | --- | --- |
| **(Intercept)** | **4.474** | **0.798** | **2.891 – 6.058** | **92.463** | **5.61** | **<.001** |
| **GROUP** | **1.168** | **0.382** | **0.405 – 1.921** | **93.323** | **3.05** | **0.003** |
| TIME | -0.221 | 0.147 | -0.517 – 0.076 | 37.982 | -1.50 | 0.141 |
| GROUP*TIME | -0.170 | 0.098 | -0.369 – 0.028 | 38.255 | -1.74 | 0.090 |
| AGE | -0.009 | 0.014 | -0.037 – 0.018 | 51.401 | -0.67 | 0.505 |
| SEX | -0.097 | 0.266 | -0.632 – 0.438 | 48.831 | -0.37 | 0.717 |

Dependent variable: *LEA*

|  | Estimate | SE | CI | *df* | t | *p* |
| --- | --- | --- | --- | --- | --- | --- |
| (Intercept) | 2.701 | 0.472 | 1.759 – 3.643 | 67.791 | 5.72 | <.001 |
| GROUP | 0.353 | 0.205 | -0.056 – 0.761 | 89.893 | 1.72 | 0.090 |
| TIME | -0.108 | 0.065 | -0.238 – 0.022 | 50.213 | -1.67 | 0.100 |
| GROUP*TIME | -0.057 | 0.043 | -0.144 – 0.030 | 50.518 | -1.32 | 0.195 |
| AGE | -0.005 | 0.009 | -0.022 – 0.013 | 50.828 | -0.51 | 0.611 |
| SEX | -0.286 | 0.172 | -0.632 – 0.060 | 49.917 | -1.66 | 0.103 |

Dependent variable: *AA*

|  | Estimate | SE | CI | *df* | t | *p* |
| --- | --- | --- | --- | --- | --- | --- |
| (Intercept) | 239.447 | 162.484 | -82.450 – 561.344 | 113.450 | 1.47 | 0.143 |
| GROUP | 64.881 | 82.866 | -98.783 – 228.544 | 158.589 | 0.78 | 0.435 |
| **TIME** | **129.685** | **34.606** | **61.482 – 197.889** | **218.487** | **3.75** | **<.001** |
| GROUP*TIME | -5.068 | 23.099 | -50.594 – 40.457 | 218.746 | -0.22 | 0.827 |
| AGE | -3.566 | 2.593 | -8.736 – 1.604 | 71.681 | -1.38 | 0.173 |
| SEX | -58.862 | 49.936 | -158.458 – 40.735 | 69.920 | -1.18 | 0.242 |

**Table S6B** Linear mixed models for repeated measures parameters with the fixed factor *GROUP*, *TIME*, and *GROUP*TIME* interactions controlled for *AGE,* *SEX, and CANNABIS USE*

Dependent variable: *OEA*

|  | Estimate | SE | CI | *df* | t | *p* |
| --- | --- | --- | --- | --- | --- | --- |
| **(Intercept)** | **1.696** | **0.436** | **0.824 – 2.569** | **58.654** | **3.89** | **<.001** |
| **GROUP** | **0.463** | **0.159** | **0.146 – 0.780** | **79.511** | **2.90** | **0.005** |
| TIME | -0.047 | 0.041 | -0.130 – 0.035 | 35.916 | -1.17 | 0.251 |
| GROUP*TIME | -0.030 | 0.027 | -0.085 – 0.025 | 36.140 | -1.12 | 0.272 |
| AGE | -0.006 | 0.008 | -0.021 – 0.009 | 50.542 | -0.81 | 0.422 |
| SEX | -0.218 | 0.139 | -0.497 – 0.060 | 49.963 | -1.58 | 0.122 |
| CANNABIS USE | 0.018 | 0.143 | -0.268 – 0.305 | 49.822 | 0.13 | 0.899 |

Dependent variable: *PEA*

|  | Estimate | SE | CI | *df* | t | *p* |
| --- | --- | --- | --- | --- | --- | --- |
| **(Intercept)** | **4.547** | **0.918** | **2.720 – 6.373** | **81.396** | **4.95** | **<.001** |
| **GROUP** | **1.148** | **0.393** | **0.368 – 1.929** | **97.003** | **2.92** | **0.004** |
| TIME | -0.220 | 0.147 | -0.518 – 0.077 | 37.922 | -1.50 | 0.141 |
| GROUP*TIME | -0.171 | 0.098 | -0.369 – 0.028 | 38.196 | -1.74 | 0.090 |
| AGE | -0.008 | 0.015 | -0.038 – 0.021 | 51.006 | -0.57 | 0.569 |
| SEX | -0.100 | 0.267 | -0.637 – 0.436 | 48.780 | -0.38 | 0.708 |
| CANNABIS USE | -0.044 | 0.274 | -0.594 – 0.507 | 48.260 | -0.16 | 0.874 |

Dependent variable: *LEA*

|  | Estimate | SE | CI | *df* | t | *p* |
| --- | --- | --- | --- | --- | --- | --- |
| **(Intercept)** | **3.080** | **0.549** | **1.982 – 4.177** | **62.945** | **5.61** | **<.001** |
| GROUP | 0.275 | 0.212 | -0.146 – 0.697 | 88.566 | 1.30 | 0.198 |
| TIME | -0.108 | 0.065 | -0.238 – 0.023 | 49.691 | -1.66 | 0.103 |
| GROUP*TIME | -0.057 | 0.043 | -0.144 – 0.030 | 49.996 | -1.32 | 0.194 |
| AGE | 0.000 | 0.009 | -0.019 – 0.018 | 50.786 | -0.03 | 0.979 |
| SEX | -0.304 | 0.170 | -0.646 – 0.038 | 49.957 | -1.79 | 0.080 |
| CANNABIS USE | -0.228 | 0.175 | -0.579 – 0.123 | 49.757 | -1.30 | 0.198 |

Dependent variable: *AA*

|  | Estimate | SE | CI | *df* | t | *p* |
| --- | --- | --- | --- | --- | --- | --- |
| **(Intercept)** | **119.697** | 180.996 | -239.308 – 478.703 | 101.978 | 0.66 | 0.510 |
| GROUP | 90.282 | 83.832 | -75.333 – 255.898 | 153.189 | 1.08 | 0.283 |
| **TIME** | **129.414** | **34.364** | **61.685 – 197.143** | **217.239** | **3.77** | **<.001** |
| GROUP*TIME | -5.084 | 22.938 | -50.293 – 40.143 | 217.501 | -0.22 | 0.825 |
| AGE | -4.955 | 2.722 | -10.381 – 0.471 | 71.602 | -1.82 | 0.073 |
| SEX | -53.431 | 49.272 | -151.698 – 44.836 | 70.072 | -1.08 | 0.282 |
| CANNABIS USE | 73.280 | 50.491 | -27.428 – 173.987 | 69.721 | 1.45 | 0.151 |

**Table S7A** Independent t-test for AUC_i_ of NAEs and AA

|  | **Controls** | **NMPOU** | **Value** | ***df*** | ***p*** |
| --- | --- | --- | --- | --- | --- |
|  | (n=29) | (n=21) |  |  |  |
| **PEA** | **-89.1 (70.6)** | **-145.0 (82.4)** | **t = 2.58** | **48** | **0.013** |
| OEA | -16.5 (26.7) | -26.1 (19.5) | t = 1.40 | 48 | 0.168 |
| LEA | -41.7 (42.2) | -50.9 (30.6) | t = 0.85 | 48 | 0.400 |
| AA | 28989.3 (13814.1) | 31597.9 (12497.5) | t = -0.69 | 48 | 0.496 |

AA: arachidonic acid, LEA: linoleoyl ethanolamide, OEA: oleoylethanolamide, PEA: palmitoylethanolamide

**Table S7B** ANCOVAs of NAEs and AA controlled for *SEX* and *AGE*

|  | **Controls** | **NMPOU** | ***F*** | ***df1*** | ***df2*** | ***p*** |
| --- | --- | --- | --- | --- | --- | --- |
|  | (n=29) | (n=21) |  |  |  |  |
| **PEA** | **-89.1 (70.6)** | **-145.0 (82.4)** | **6.4** | **1** | **45** | **0.015** |
| OEA | -16.5 (26.7) | -26.1 (19.5) | 1.5 | 1 | 45 | 0.227 |
| AA | 28989 (13814) | 31598 (12498) | 1.1 | 1 | 45 | 0.291 |
| LEA | -41.7 (42.2) | -50.9 (30.6) | 0.6 | 1 | 45 | 0.437 |

AA: arachidonic acid, LEA: linoleoyl ethanolamide, OEA: oleoylethanolamide, PEA: palmitoylethanolamide

**Table S7C** ANCOVAs of NAEs and AA controlled for *SEX*, *AGE*, and *CANNABIS USE*

|  | **Controls** | **NMPOU** | ***F*** | ***df1*** | ***df2*** | ***p*** |
| --- | --- | --- | --- | --- | --- | --- |
|  | (n=29) | (n=21) |  |  |  |  |
| **PEA** | **-89.1 (70.6)** | **-145.0 (82.4)** | **5.1** | **1** | **45** | **0.029** |
| AA | 28989 (13814) | 31598 (12498) | 1.9 | 1 | 45 | 0.176 |
| OEA | -41.7 (42.2) | -50.9 (30.6) | 1.0 | 1 | 45 | 0.315 |
| LEA | -16.5 (26.7) | -26.1 (19.5) | 0.04 | 1 | 45 | 0.849 |

AA: arachidonic acid, LEA: linoleoyl ethanolamide, OEA: oleoylethanolamide, PEA: palmitoylethanolamide

**
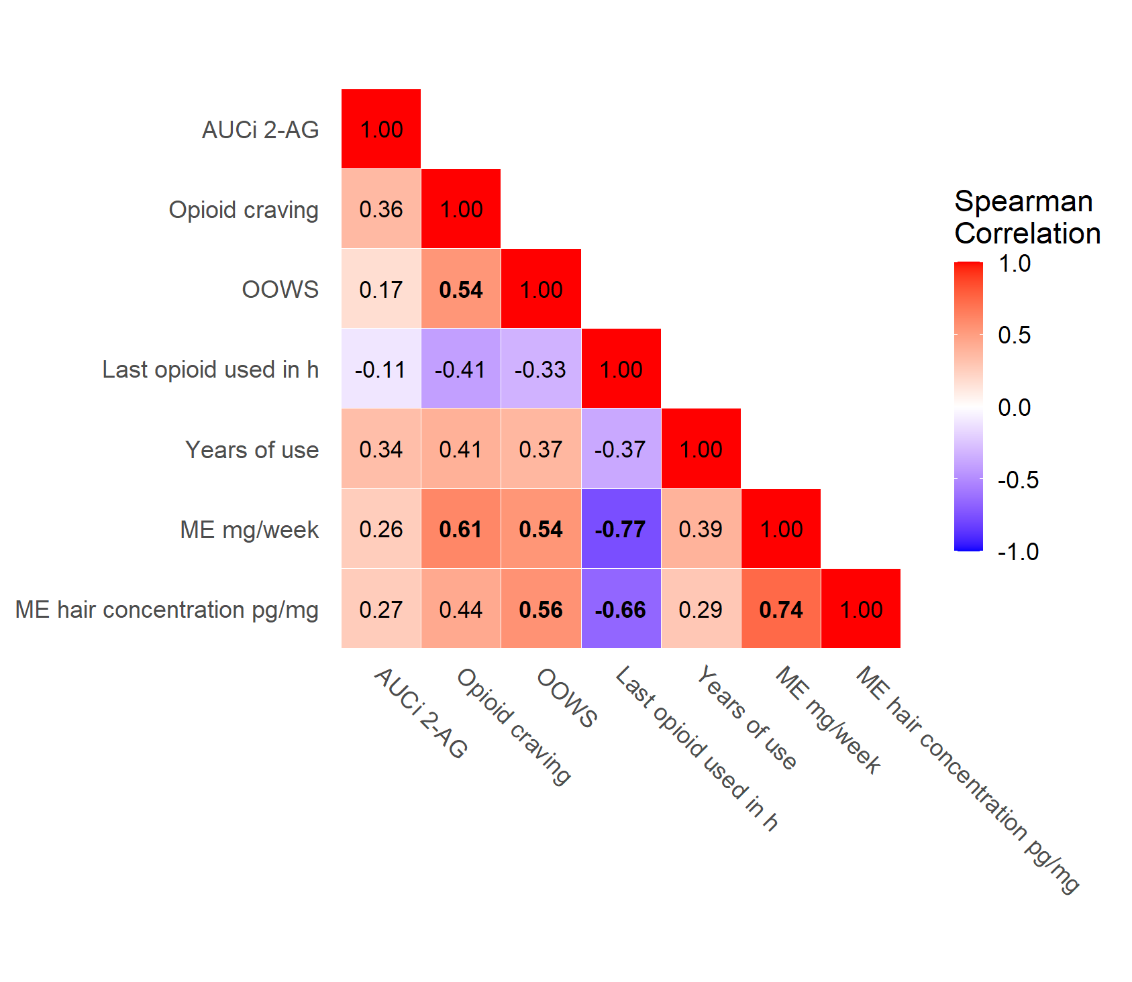
**

**Fig. S1** Spearman’s rank correlation between AUC_i_ 2-AG and opioid use variables. Heat matrix of correlation coefficients with significant correlations shown in bold (*p*<.05) within the NMPOU group. AUC_i_: area under the curve with respect to increase, ME: morphine equivalence, OOWS: objective opioid withdrawal symptoms, 2-AG: 2-arachidonylglycerol

**
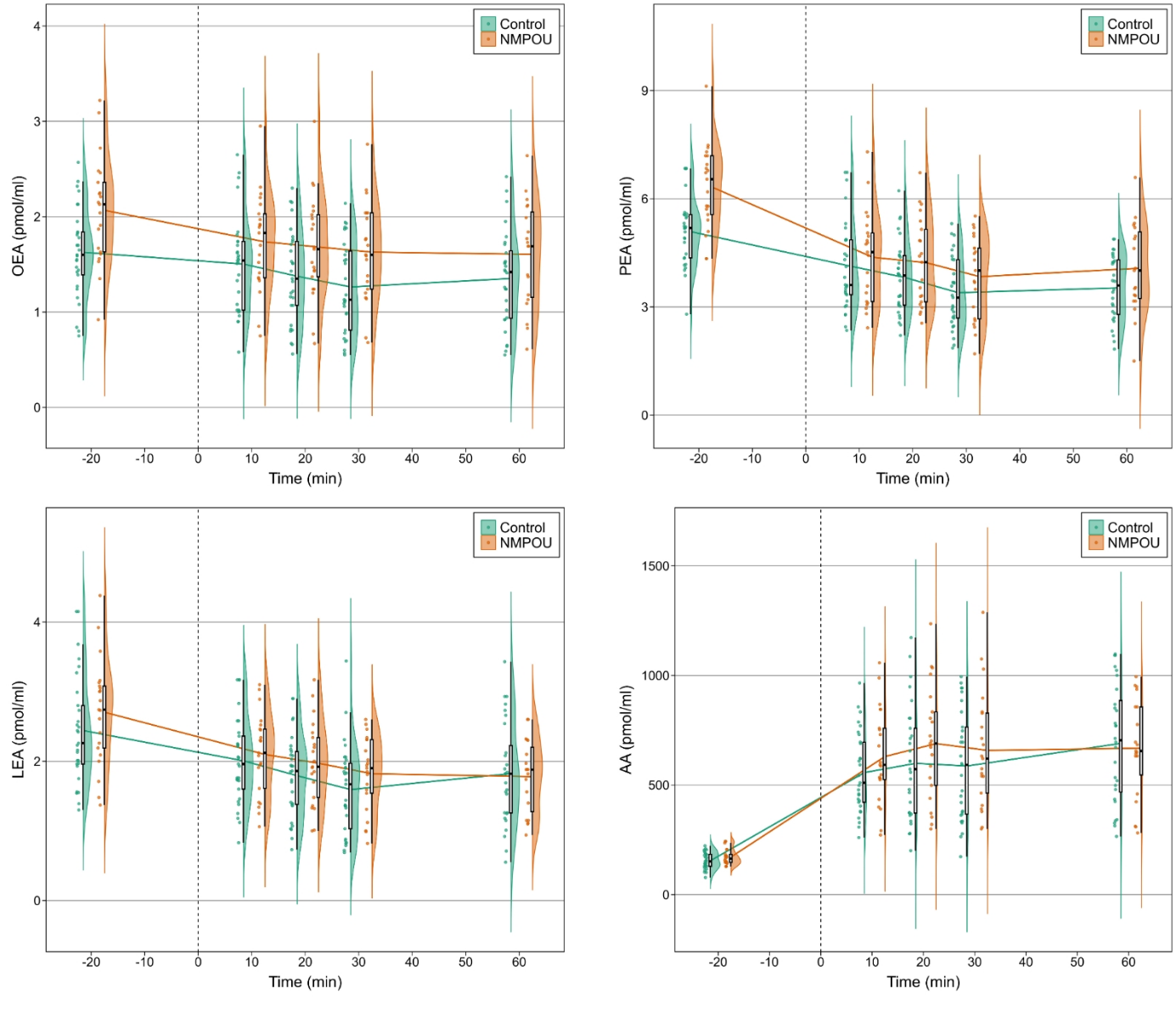
Fig. S2** Timeline plots with individual data points showing mean plasma concentrations of endocannabinoids and related lipids at baseline and 10, 20, 30, and 60 minutes following the Cyberball task in individuals with NMPOU and healthy controls. Lines indicate group means with half-violin plots showing group distribution and boxplots including median and interquartile range (IQR), with whiskers extending to the most extreme value within 1.5×IQR of the lower and upper quartiles at each timepoint. The dotted line indicates the start of the Cyberball task. AA: arachidonic acid, LEA: linoleoyl ethanolamide, OEA: oleoylethanolamide, PEA: palmitoylethanolamide


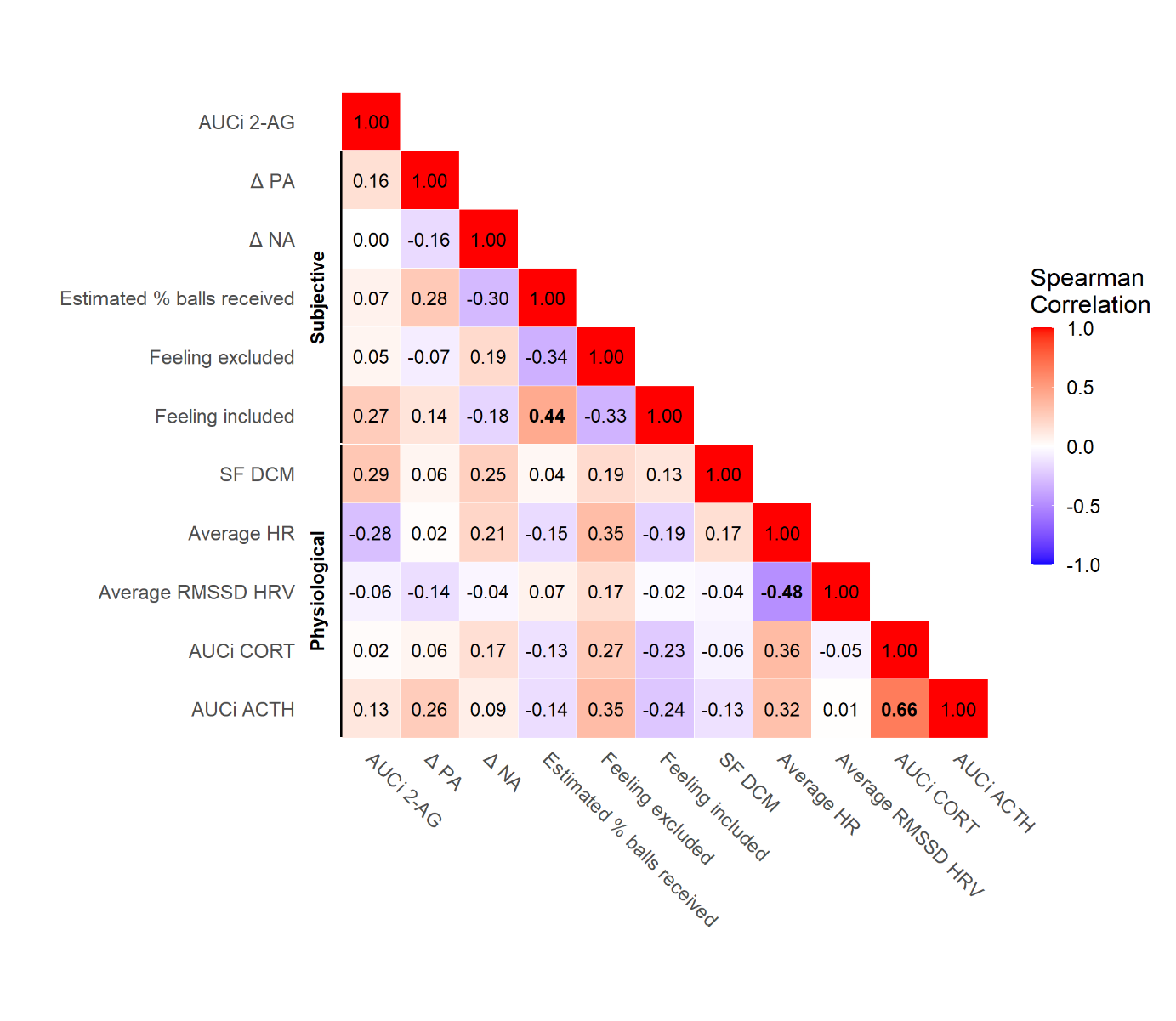


**Fig. S3A** Within control group: Spearman’s rank correlation between AUC_i_ 2-AG, subjective social exclusion variables, and physiological stress variables. Heat matrix of correlation coefficients with significant correlations shown in bold (*p*<.05) within the control group. ACTH: adrenocorticotropic hormone, AUC_i_: area under the curve with respect to increase, CORT: cortisol, DCM: dynamic casual modeling, HR: heart rate, HRV: heart rate variability, NA: negative affect, PA: positive affect, RMSSD: root mean square of successive differences, SF: spontaneous fluctuation, 2-AG: 2-arachidonylglycerol


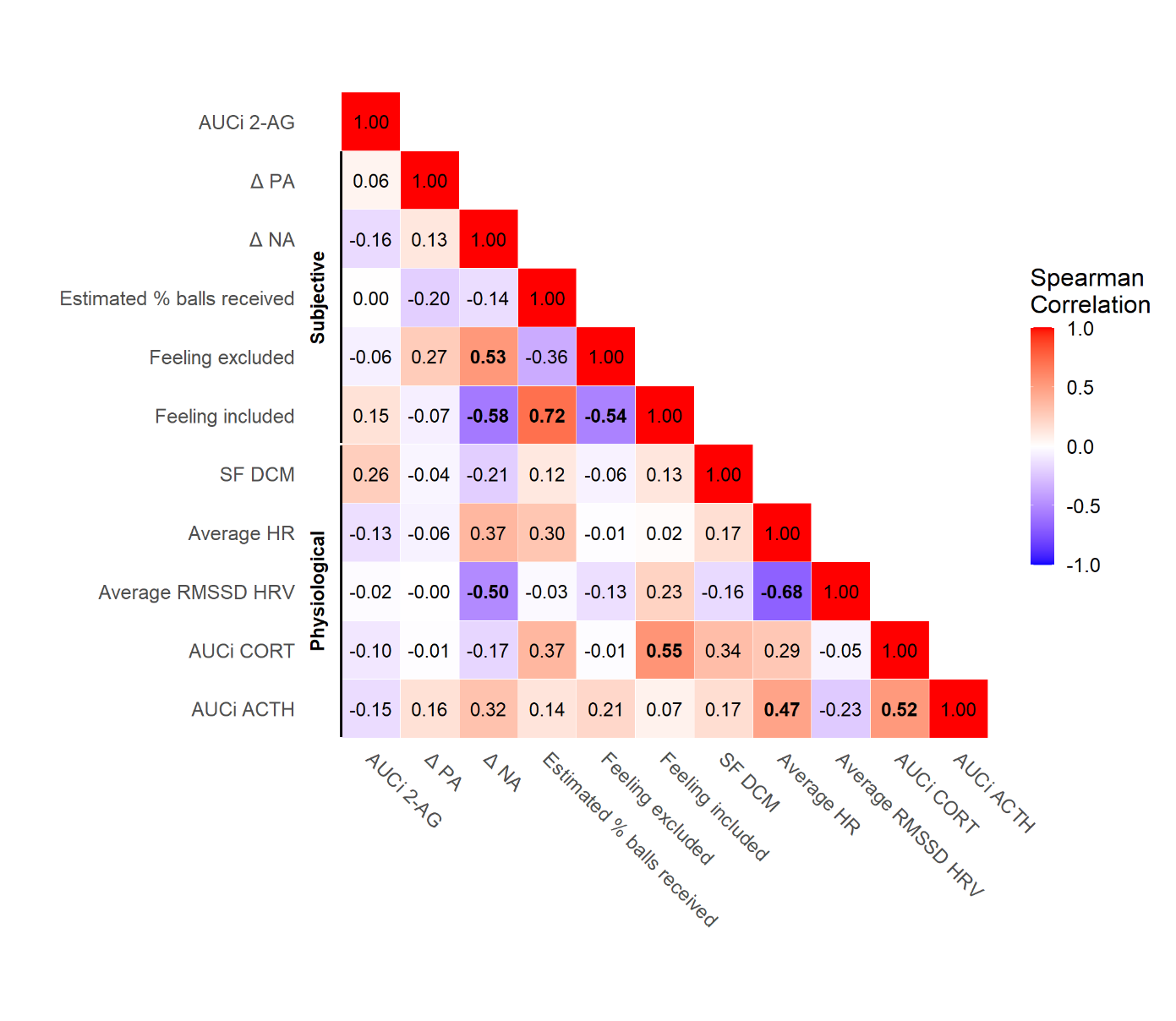


**Fig. S3B** Within NMPOU group: Spearman’s rank correlation between AUC_i_ 2-AG, subjective social exclusion variables, and physiological stress variables. Heat matrix of correlation coefficients with significant correlations shown in bold (*p*<.05) within the NMPOU group. ACTH: adrenocorticotropic hormone, AUC_i_: area under the curve with respect to increase, CORT: cortisol, DCM: dynamic casual modeling, HR: heart rate, HRV: heart rate variability, NA: negative affect, PA: positive affect, RMSSD: root mean square of successive differences, SF: spontaneous fluctuation, 2-AG: 2-arachidonylglycerol

References

1 Reynoso-Moreno I, Tietz S, Vallini E, Engelhardt B, Gertsch J, Chicca A. *Selective Endocannabinoid Reuptake Inhibitor WOBE437 Reduces Disease Progression in a Mouse Model of Multiple Sclerosis.* ACS Pharmacol Transl Sci. 2021;4(2):765-79.

2 Hillard CJ. *Circulating Endocannabinoids: From Whence Do They Come and Where are They Going?* Neuropsychopharmacology. 2018;43(1):155-72.
